# Estimating Soil Arsenic Contamination by Integrating Hyperspectral and Geochemical Data with PCA and Optimizing Inversion Models

**DOI:** 10.3390/s25226857

**Published:** 2025-11-10

**Authors:** Fei Guo, Zhen Xu, Honghong Ma, Xiujin Liu

**Affiliations:** 1 Institute of Geophysical & Geochemical Exploration, Chinese Academy of Geological Sciences, Langfang 065000, China; flynn1991@sina.cn (F.G.); mahonghong@mail.cgs.gov.cn (H.M.); liuxiujin@mail.cgs.gov.cn (X.L.); 2 Key Laboratory of Geochemical Cycling of Carbon and Mercury in the Earth’s Critical Zone, Chinese Academy of Geological Sciences, Langfang 065000, China; 3 Geochemical Research Center of Soil Quality, China Geological Survey, Langfang 065000, China; 4 College of Information Science and Engineering, Hohai University, Nanjing 211100, China

**Keywords:** hyperspectral, soil arsenic contamination, PCA, multi-source data fusion

## Abstract

**Highlights:**

**What are the main findings?**

- This study proposed a novel inversion method based on the fusion of geochemical data and PCA-dimensionality-reduced spectral data.
- This study demonstrated the superior performance of the Random Forest model on the fused data.

**What is the implication of the main finding?**

- This study innovatively integrates direct and precise geochemical data with macroscopic and continuous spectral data. By applying Principal Component Analysis to reduce the dimensionality and noise of the high-dimensional spectral data, core features are effectively extracted, resulting in a fused dataset with more comprehensive information. This method overcomes the limitations of using a single data source and significantly improves the inversion accuracy for mapping the spatial distribution of soil arsenic.
- Among various machine learning models, this study conclusively verifies the exceptional effectiveness of the Random Forest algorithm in processing the multi-source fused data. The model proficiently captures the complex non-linear relationships between arsenic content and multi-source environmental features. Its inherent resistance to overfitting and capability for feature importance assessment provide a reliable tool for high-precision arsenic inversion and pollution mechanism analysis.

**Abstract:**

Soil arsenic (As) contamination presents serious threats to ecosystems and human health, necessitating the development of accurate and efficient monitoring techniques. This study introduces a novel multi-source data fusion approach to enhance the hyperspectral inversion of soil arsenic concentrations by integrating dimensionality-reduced spectral data with soil components significantly correlated with arsenic (e.g., Cd, Cr, Cu, Ni, Pb, Zn, S, and total Fe_2_O_3_(T-Fe_2_O_3_)). Principal Component Analysis (PCA) was utilized to reduce the dimensionality of hyperspectral data, effectively addressing issues of collinearity and redundancy while preserving critical spectral information. The performances of three models, namely Partial Least Squares Regression (PLSR), Artificial Neural Networks (ANN), and Random Forest (RF), were assessed under four input variable combinations: (1) original spectral data, (2) original spectral data with soil components, (3) PCA dimensionality-reduced spectral data, and (4) PCA dimensionality-reduced spectral data combined with soil components. The results demonstrated that the RF model, when applied to the multi-source data of PCA-reduced spectra and soil components, achieved the highest inversion accuracy with an R^2^ value of 0.86, significantly outperforming the PLSR model (R^2^ = 0.75). This study underscores the effectiveness of enhancing model performance and highlights the superior capability of the RF model in handling complex, high-dimensional datasets. The findings of soil arsenic estimation provide theoretical foundation for optimizing hyperspectral remote sensing technology in monitoring soil heavy metal contamination and establishing a robust framework for future research and practical applications in environmental science.

## 1. Introduction

Arsenic (As), a toxic metalloid prevalent in natural environments, poses severe threats to ecosystems and human health through excessive accumulation [1,2]. Though it is not a true heavy metal [3,4], arsenic exhibits high toxicity, environmental mobility, and behavioral similarities to heavy metals [1]. Consequently, arsenic is generally termed a “pseudo-heavy metal” or “toxic element” and is systematically studied alongside heavy metals in environmental science and pollution management [5,6]. Soil arsenic contamination, driven by its persistence, bioaccumulation, and toxicity, poses significant risks to ecological integrity and public health [2,7]. From this point of view, accurate monitoring and estimation of soil arsenic concentrations are crucial for advancing environmental governance, pollution mitigation, and food safety protocols [8].

Traditional soil heavy metal detection employs field sampling with laboratory chemical analysis, supplemented by geostatistical interpolation to map spatial contamination patterns [9,10]. While offering high precision, these methods face practical constraints including labor intensity, time consumption, and elevated operational costs [11]. By contrast, Visible and Near-Infrared Reflectance (VNIR) hyperspectral spectroscopy presents a transformative alternative through its rapid, cost-effective, and non-destructive advantages [12]. Thus, the VNIR hyperspectral spectroscopy technique can capture high-resolution quasi-continuous spectral data, enabling comprehensive characterization of soil composition and facilitating efficient heavy metal detection [13,14]. Nevertheless, three inherent data challenges-such as high dimensionality, spectral collinearity, and information redundancy-often degrade model performance through overfitting and reduced inversion accuracy, hindering its practical application [15,16]. Therefore, developing optimized data processing workflows and enhanced modeling frameworks remains critical for advancing hyperspectral applications in soil pollution monitoring.

Beyond Principal Component Analysis (PCA), common dimensionality reduction methods include Linear Discriminant Analysis (LDA)—a supervised linear approach, Locally Linear Embedding (LLE), and t-distributed Stochastic Neighbor Embedding (t-SNE) for non-linear scenarios, Auto encoders (AE) driven by deep learning, and Kernel Principal Component Analysis (KPCA)—a non-linear extension of PCA. While each of these methods has its own strengths (e.g., LDA excels at classification tasks, t-SNE is adept at preserving local clustering structures, and AE can adapt to complex data distributions), they all suffer from notable drawbacks compared to PCA: generally, they face higher computational complexity and stronger reliance on parameters or additional information (such as labels and large sample sizes). In contrast, PCA boasts advantages of linear efficiency, stability, and reliability, as well as strong interpretability, making it more versatile across various application scenarios [17]. Principal Component Analysis (PCA) demonstrates substantial advantages in hyperspectral inversion through effective dimensionality reduction [18,19]. By compressing high-dimensional spectral data into a few principal components (typically accounting for >95% cumulative variance), PCA eliminates inter-band collinearity, preserves critical spectral features, and suppresses noise, thereby enhancing model accuracy and computational efficiency [19,20]. Empirical studies validate PCA’s utility in soil heavy metal inversion, where it reduces hundreds of spectral bands to 3–5 interpretable components while maintaining diagnostic spectral signatures [18,21,22]. Beyond terrestrial applications, PCA has proven effective in aquatic hyperspectral monitoring, particularly for quantifying suspended sediments and chlorophyll-a concentrations [23,24]. However, the linear transformation mechanism of PCA struggles with non-linear spectral response patterns inherent in complex soil matrices. Recent advancements propose synergistically integrating PCA with machine learning architectures and multi-source geospatial data to address this limitation and expand its operational scope [25].

While hyperspectral data can partially characterize soil properties, its inherent limitations constrain its ability to meet the requirements for high-precision inversion [26,27]. The VNIR data mainly captures the spectral response properties of the soil surface [28], whereas the distribution and concentration of soil heavy metals (such as arsenic, cadmium, and lead) are influenced by a variety of environmental factors, including soil components (such as organic matter, iron oxides, and clay minerals), pH value, redox conditions, and human activities [5,29]. Soil constituents not only provide a critical environmental context but also compensate for the shortcomings of spectral data in characterizing the internal chemical properties of soil [30]. For example, iron oxides (such as hematite and goethite) have a strong adsorption capacity for heavy metals like arsenic, and their content is closely related to heavy metal concentrations. Similarly, sulfides can form insoluble compounds with arsenic under reducing conditions, thereby affecting the mobility and bioavailability of arsenic [31,32]. In this view, exclusive reliance on spectral inputs risks omitting environmentally mediated mechanisms, ultimately compromising inversion accuracy.

The critical role of inversion models in predicting soil heavy metal concentrations necessitates judicious model selection and optimization to ensure prediction accuracy, reliability, and operational feasibility [18,33]. Different inversion models demonstrate distinct performances when it comes to processing hyperspectral data and estimating soil compositional parameters [26]. Linear inversion models like Partial Least Squares Regression (PLSR) excel in handling high-dimensional datasets through latent variable extraction, effectively mitigating spectral collinearity in strongly linear relationships [34,35]. However, their capacity to interpret complex nonlinear interactions remains constrained. Conversely, nonlinear models, including but not limited to Random Forest (RF), Support Vector Machine (SVM), and Artificial Neural Network (ANN), can better capture the complex non-linear relationships between spectral data and soil heavy metal concentrations [36,37,38]. Among the non-linear models, the RF model stands out in multi-source data fusion, capable of combining spectral data and soil component information to significantly improve inversion accuracy while being highly robust to noise and outliers [39]. The SVM leverages kernel functions to process nonlinear patterns, making it suitable for limited-sample scenarios [40]. The ANN, despite superior nonlinear fitting capabilities, demands high-quality training data, large sample sizes, and careful regularization to prevent overfitting [41,42]. Accordingly, optimal model selection must balance data characteristics (dimensionality, sample size, noise levels), research objectives, and computational resources.

To tackle the aforementioned challenges and limitations, this study introduces an innovative multi-source fusion framework integrating dimensionality-reduced hyperspectral features with arsenic-associated soil constituents. PCA-transformed spectral data and geochemically active soil components (e.g., iron oxides, organic matter) substantially enhance the accuracy and operational robustness of the arsenic inversion model. Systematic performance benchmarking evaluated three machine learning architectures in terms of PLSR, RF, and ANN under different input variable combinations to determine the optimal modeling strategy.

The main contributions of this study are threefold: (1) developing a multi-source data fusion framework to deal with hyperspectral data constraints in heavy metal inversion; (2) applying PCA for optimized spectral dimensionality reduction while retaining essential spectral information; and (3) comprehensively evaluating model performance through comprehensive metrics to identify the optimal model for inverting the soil arsenic concentration. This study not only provides a scientific basis and technical support for hyperspectral remote sensing monitoring of soil heavy metal pollution but also offers valuable insights into multi-source data fusion and model optimization. The proposed framework can be extended to similar toxic elements and other environmental monitoring scenarios, contributing to the advancement of remote sensing technology in the field of environmental science.

## 2. Materials and Methods

### 2.1. Description of the Study Area

**Figure 1:**
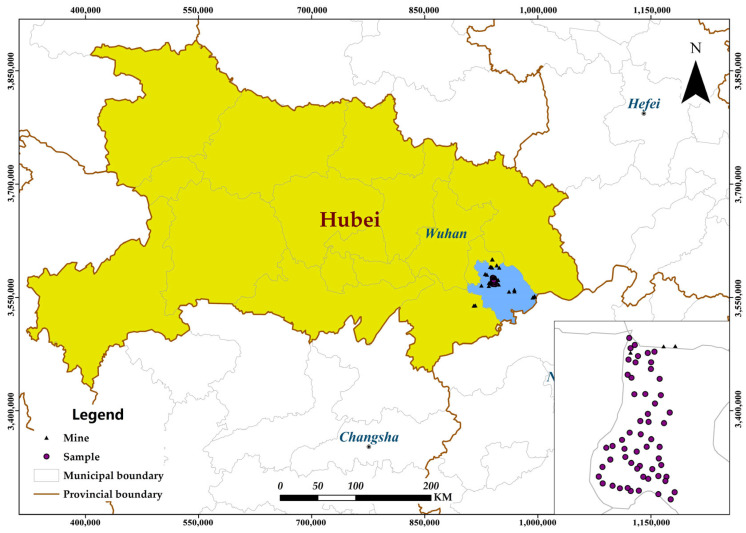
Overview of the study area and sampling points.

The study area is located in Daye City, Hubei Province, whose topography exhibits a distinctive pattern: ascending southward, and descending northward, while maintaining a relatively flat and uniform terrain along the east–west axis [43]. Geographically, the area extends between 114°31′ E to 115°20′ E and 29°40′ N to 30°15′ N (Figure 1). The terrain consists primarily of hills, mountains, and plains, with elevations ranging from 120 to 200 m above sea level [18]. Daye City possesses abundant mineral resources with significant diversity. However, the extensive mining and smelting operations in the region have inevitably led to soil contamination in the surrounding areas [44,45].

### 2.2. Soil Sampling and Data Preprocessing

As shown in Figure 1, 56 soil samples were collected across the study area. The sampling locations were strategically distributed to ensure comprehensive coverage of diverse land use patterns and soil conditions. Surface soil samples (0–20 cm depth) were extracted at each sampling point using a standardized stainless steel soil sampler, with each sample weighing more than 1000 g [18,46]. Sample preparation followed a standardized protocol: air-drying, grinding, and sieving through a 2 mm (10-mesh) nylon screen [47]. Following preparation, each sample was divided into two portions: one designated for chemical analysis in the laboratory, and the other allocated for spectroscopic reflectance measurements.

#### 2.2.1. Laboratory Analysis

Laboratory analysis was conducted on the collected soil samples to determine the actual concentrations of heavy metals and other soil elements, establishing ground truth data for model validation. The soil’s arsenic concentrations were specifically measured using determination of arsenic, antimony, and bismuth by hydride atomic fluorescence spectrometry. The analytical methods for other elements (e.g., Cd, Cr, Cu, Hg…) are detailed in Table 1. In this study, quality control measurements, including the employment of standard reference materials and duplicate samples, were implemented throughout the laboratory analysis process to ensure analytical accuracy and data reliability.

#### 2.2.2. Hyperspectral Data Acquisition

The soil hyperspectral data were measured using an ASD FieldSpec4 spectrometer (Analytical Spectral Device, Inc., Longmont, CO, USA) with a spectral range of 350–2500 nm (Visible and Near-Infrared Reflectance (VNIR) hyperspectral spectroscopy) [34]. The instrument features varying sampling intervals: 1.4 nm for the 350–1100 nm range and 2 nm for the 1000–2500 nm range [48]. Through resampling, the final output provided 2151 spectral bands at uniform 1 nm intervals.

The spectral measurements were conducted under controlled laboratory conditions. A 50 W halogen lamp served as the illumination source in a dark room environment. The samples were positioned at a 15° angle and 50 cm from the light source to prevent shadowing effects. The optical probe is mounted about 7 cm above the sample surface. To ensure the measurement stability, the spectroradiometer was preheated for 30 min before the data collection. Prior to the first measurement, the instrument was calibrated using a standard white BaSO_4_ reference panel [49]. Each soil sample was homogeneously distributed in a Petri dish, from which 10 consecutive spectral measurements were acquired. Finally, the spectral reflectances with wavelength ranges of 350–399 nm and 2450–2500 nm were excluded from this analysis due to their low signal-to-noise ratio (SNR) [18,48,50].

**Figure 2:**
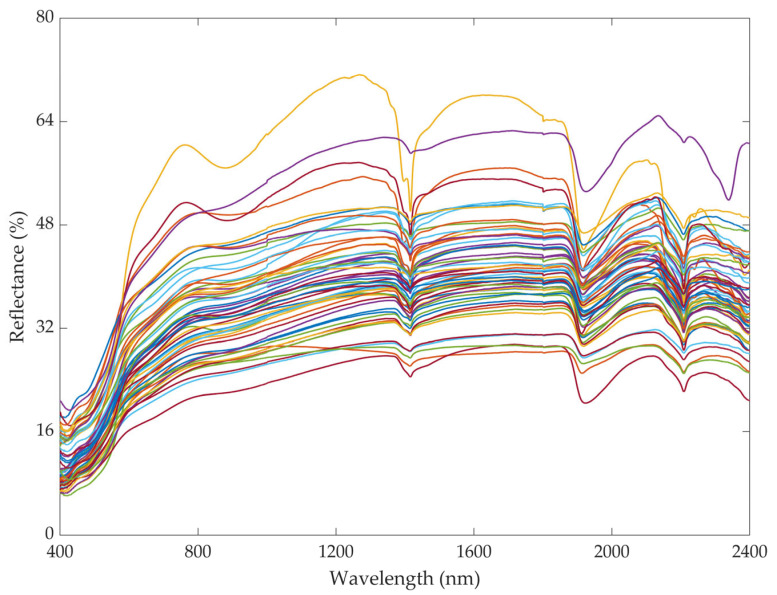
The original spectral reflectance.

As presented in Figure 2, the spectral reflectance curves exhibit three distinct absorption features centered at the wavelengths of approximately 1400 nm, 1900 nm, and 2200 nm. These absorption valleys, particularly pronounced after spectral preprocessing, are characteristic of soil clay mineral compositions. The presence and depth of these absorption features directly correlate with the soil’s mineralogical properties [51].

### 2.3. Model Input Variable Filtering

#### 2.3.1. Principal Component Analysis

PCA is an effective dimensionality reduction technique optimized for feature extraction and visualization of high-dimensional data [52,53]. The method employs a systematic process of data standardization, covariance matrix computation, eigenvalue decomposition, and projection to effectively reduce high-dimensional data into a lower-dimensional space while preserving essential information characteristics [54]. The fundamental principle of PCA operates by identifying directions of maximum variance (principal components) within the dataset [55]. These principal components are orthogonal vectors arranged in descending order of explained variance. Generally, the first principal component captures the highest variance, followed by subsequent components with progressively decreasing variance contributions. Numerous studies have demonstrated that PCA plays a significant role in improving model inversion accuracy for heavy metal estimation based on hyperspectral reflectance data. The method’s effectiveness in reducing data dimensionality while maintaining crucial spectral information has made it a fundamental processing step in hyperspectral data analysis [56,57].

#### 2.3.2. Correlation Analysis

Correlation analysis is a statistical methodology for quantifying the magnitude and directionality of relationships across different variables [58]. Correlation analysis identifies linear relationships crucial for feature selection in modeling [59,60]. The metric used to measure the linear relationship between variables ranges from −1 to +1, where a value near −1 represents a strong negative correlation, while a value near 1 indicates a strong positive correlation [61]. The Pearson correlation coefficient, specifically, is widely used to identify features that exhibit strong correlations with target variables. This coefficient serves as a crucial tool for selecting input variables in regression analysis and classification models. In this research, we employ correlation analysis to identify soil properties that demonstrate significant relationships with arsenic contamination levels. By integrating these correlated soil properties with dimensionally reduced hyperspectral data, we aim to enhance both the prediction accuracy and practical applicability of the arsenic contamination model.

### 2.4. Model Construction and Validation

#### 2.4.1. Modeling Method

Various inversion models were employed in this study, wherein the PLSR is a highly effective multivariate statistical modeling approach specifically designed for handling high-dimensional data, such as hyperspectral measurements, and addressing multicollinearity issues [62]. Originally developed by Wold et al. in 1983 [18], PLSR has become one of the most widely adopted linear modeling techniques. The method operates by extracting latent variables that link independent variables (hyperspectral bands) with dependent variables (heavy metal concentrations) to construct a linear regression model [18,63]. The fundamental principle of PLSR lies in its ability to maximize covariance between independent and dependent variables during dimensionality reduction, thereby efficiently capturing essential data patterns. PLSR demonstrates several key advantages: it effectively manages high-dimensional datasets, resolves multicollinearity issues, and maintains robustness against noise and outliers in the data structure [64]. In hyperspectral inversion applications, PLSR has proven particularly valuable for estimating soil heavy metal concentrations.

ANN has emerged as a powerful tool for predicting heavy metal concentrations from hyperspectral data due to its exceptional nonlinear modeling capabilities [65,66]. The architecture of ANN, comprising multiple neural layers and nonlinear activation functions (ReLU, Sigmoid), effectively captures nonlinear relationships between the hyperspectral signatures and heavy metal concentrations. The key strength of ANN lies in its adaptive learning mechanism and generalization ability. This allows for automatic feature extraction from large datasets and optimal parameter adjustment through training [67]. In hyperspectral applications, ANN processes spectral bands as input nodes and produces heavy metal concentrations as output, with nonlinear transformations occurring through hidden layers. ANN demonstrates superior performance compared to traditional linear models, particularly in handling nonlinear relationships and high-dimensional hyperspectral data. The model’s accuracy can be further enhanced by incorporating additional environmental variables, such as soil properties and topographic data. However, ANN implementation faces several challenges: it requires large datasets and careful tuning to prevent overfitting, and complex hyperparameter optimization. These limitations can be effectively addressed through cross-validation, regularization, and early stopping techniques [68]. Overall, ANN holds broad application prospects in the hyperspectral inversion of heavy metals, providing reliable technical support for environmental monitoring and pollution control.

RF represents an ensemble learning approach that has proven highly effective for heavy metal concentration estimation using hyperspectral data [69,70]. The method operates by constructing multiple decision trees and aggregating their predictions, demonstrating robust performance in handling high-dimensional spectral data [69]. The advantages of RF include automatic feature selection capability, efficient dimensionality reduction, and strong resilience to noise and outliers in hyperspectral measurements. The ensemble nature of RF enhances prediction accuracy and stability compared to single-model approaches, particularly in soil heavy metal estimation [70]. Additionally, the method requires minimal parameter tuning and offers straightforward implementation and interpretation. However, RF may face computational challenges with high-dimensional datasets and show limitations with small sample sizes, which can be addressed through strategic feature selection and hyperparameter optimization procedures. Overall, RF has broad application potential in the hyperspectral inversion of heavy metals, providing an efficient and reliable tool for environmental monitoring and pollution assessment.

#### 2.4.2. Model Validation

The models are validated using cross-validation techniques to ensure their generalizability and robustness. The performance of the models is evaluated using metrics such as the coefficient of determination (R^2^), root mean square error (RMSE), and Residual Prediction Deviation (RPD) [71].

(1) $$R^{2}=1-\frac{\sum_{i=1}^{n}{\left(y_{i}-{\hat{y}}_{i}\right)}^{2}}{\sum_{i=1}^{n}{\left(y_{i}-{\bar{y}}_{i}\right)}^{2}}$$

(2) $$RMSE=\sqrt{\frac{1}{n}\sum_{i=1}^{n}{\left({\hat{y}}_{i}-y_{i}\right)}^{2}}$$

(3) $$RPD=\frac{S.D.}{RMSE}$$

where n represents the number of samples; ${\bar{y}}_{i}$ and ${\hat{y}}_{i}$ denote the measured and predicted values in the validation set, respectively; ${\bar{y}}_{i}$ is the mean of the measured values; and S.D. stands for the standard deviation.

A robust model is typically defined by high R^2^ and RPD values coupled with a low RMSE. R^2^ and RPD serve as key metrics to evaluate the accuracy of inversion performance. In contrast, RMSE is affected by the range of the measured values. The classification of RPD values is as follows: an RPD greater than 2.0 signifies excellent inversion performance; an RPD between 1.4 and 2.0 indicates the model’s ability to distinguish between high and low values; while an RPD below 1.4 denotes poor inversion performance [48,72].

### 2.5. Data Treatment

When screening independent variables, the following steps were carried out: first, Principal Component Analysis (PCA) was used to reduce the dimensionality of the spectral data (Python 3.7.2 software, Wilmington, DE, USA) for extracting principal components; meanwhile, correlation analysis was applied to analyze the soil components related to the target variable (SPSS 27.0), and variables with high correlation were selected as another part of the input variables for the model. Finally, PLSR, ANN, and RF models were used to estimate the content of arsenic (Python 3.7.2). In addition, the data statistical work involved in this study was completed using SPSS 27. All maps were generated using ArcMap 10.2 within the GIS software (ArcGIS 10.2, Esri, Redlands, CA, USA). The spectral curve graph was plotted using MATLAB Version 2013b (Matlab Inc., Natick, MA, USA).

## 3. Results

### 3.1. Arsenic Contamination of Soil

In this study, 56 soil samples were collected and divided into 38 calibration samples and 18 validation samples, maintaining an approximate 7:3 ratio [18]. Table 2 summarizes the arsenic concentrations across the samples, including the mean, maximum, minimum, standard deviation (SD), and coefficient of variation (CV). The results revealed that the average arsenic concentration in the study area was 23.01 mg/kg, with a maximum concentration reaching 57.08 mg/kg. Compared to the natural background value of 11.2 mg/kg for soil arsenic concentration, as published by the China National Environmental Monitoring Centre, 48 sample points exceeded this threshold. Notably, the maximum arsenic concentration was over four times the natural background value, indicating severe arsenic contamination in the region.

Additionally, Table 2 demonstrated significant spatial heterogeneity in the distribution of arsenic concentrations. While some areas showed minimal arsenic contamination, others were likely subjected to severe pollution. The coefficients of variation (CV) for all samples exceeded 0.36 [18], further corroborating the pronounced spatial variability of arsenic concentrations. This finding suggests the potential presence of point source pollution within the study area. Consequently, there is an urgent need to develop an accurate inversion model capable of monitoring arsenic pollution on a macro scale using hyperspectral remote sensing technology.

### 3.2. Statistical Results of Soil Properties

In this study, alongside measuring arsenic contamination in the 56 soil samples, other soil components and properties potentially associated with arsenic were analyzed. The statistical results of these measurements are summarized in Table 3. The findings revealed that the average soil pH in the study area was 6.05 ± 1.23, with 66.1% of the sampling points exhibiting pH values below 6.5. This indicates that the surface soil in the study area is predominantly acidic. Additionally, the average soil organic matter (SOM) content was slightly lower than the average SOM level of surface soil in Hubei Province, which is 56.23 mg·kg^−1^ [73].

### 3.3. Filtering of Model Input Variables

#### 3.3.1. Soil Components Associated with Arsenic

**Figure 3:**
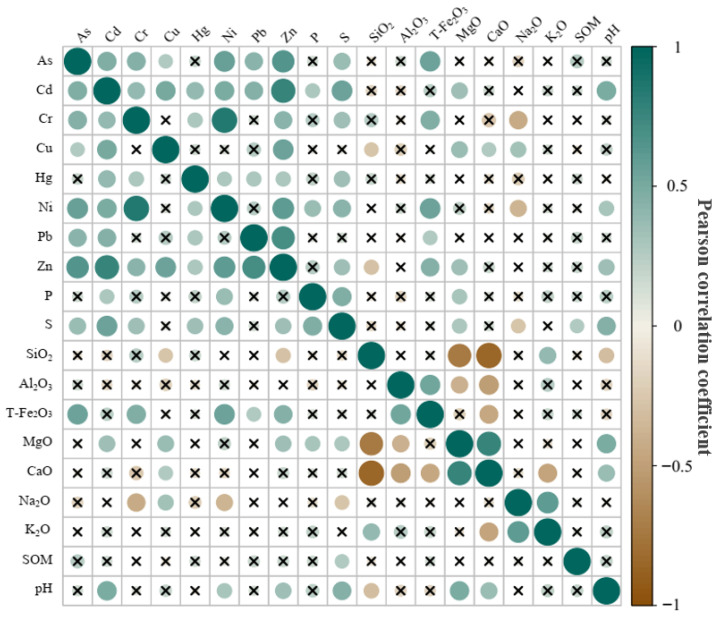
Results of correlation analysis of soil components related to arsenic elements.

In this study, Pearson’s correlation analysis was conducted to evaluate the relationships between arsenic concentrations and selected soil parameters, including heavy metals, pH, and potential arsenic-associated soil components. The objective was to identify key variables significantly influencing arsenic concentrations for inclusion as additional input variables in the inversion model. As shown in Figure 3, the analysis revealed strong correlations between arsenic and several heavy metals, specifically Cd, Cr, Cu, Ni, Pb, and Zn, with the exception of mercury (Hg). Additionally, significant correlations were identified between arsenic and sulfur (S) as well as total iron oxides (T-Fe_2_O_3_). These relationships are likely attributable to synergistic interactions among heavy metals, competitive adsorption processes, and the high adsorption capacity of iron oxides for arsenic species [74].

Figure 3 shows the pairwise correlations between elements. Green indicates a positive correlation, with darker shades representing a more significant positive correlation; yellow indicates a negative correlation, with darker shades representing a more significant negative correlation. Additionally, the size of the circle is proportional to the color intensity, both of which denote a stronger correlation. In the figure, all non-significant correlations between elements are marked with an “✕”. Based on these findings, the variables Cd, Cr, Cu, Ni, Pb, Zn, S, and T-Fe_2_O_3_ were incorporated into the model as auxiliary input parameters to improve the accuracy and reliability of arsenic concentration inversion. This approach effectively captures the complex interactions between arsenic and associated soil components while enhancing the data framework for hyperspectral remote sensing applications in monitoring soil heavy metal contamination. Consequently, this methodology contributes to advancing the technical robustness of geospatial pollution assessments.

#### 3.3.2. Spectral Dimensionality Reduction Using PCA

In this study, PCA was employed to reduce the dimensionality of the original spectral data by selecting principal components with eigenvalues greater than 1. These components were subsequently used as selected input variables for the arsenic contamination inversion model. The dimensionality reduction results, detailed in Table 4, indicate that 7 principal components have eigenvalues exceeding 1, with a cumulative contribution rate of 99.9%. This high cumulative contribution rate demonstrates that these components effectively preserve the vast majority of the information contained in the original spectral data.

By implementing PCA, data dimensionality was significantly reduced, leading to a decrease in model complexity. Simultaneously, redundant information and noise in the spectral data were eliminated, thereby enhancing the model’s computational efficiency and prediction accuracy. The ability of the selected principal components to adequately represent the features of the original spectra establishes a robust data foundation for subsequent arsenic contamination inversion modeling. These findings underscore the effectiveness of PCA in dimensionality reduction and information extraction from spectral data, providing essential support for the utilization of hyperspectral remote sensing technology in monitoring soil heavy metal pollution.

### 3.4. Performance of Inversion Models

In this study, four input variable combinations were designed for arsenic concentration inversion modeling: (1) original spectral data; (2) original spectral data combined with soil components significantly correlated with arsenic, as determined through correlation analysis; (3) principal components derived from dimensionality reduction; and (4) principal components combined with soil components significantly correlated with arsenic, as identified through correlation analysis. To evaluate the performance of these combinations, both linear models and nonlinear models were employed for inversion modeling. In addition, a scatter plot was drawn using 18 samples from the validation set, which clearly reflects the correlation between the predicted values and the measured values.

By comparing the performance of different input variable combinations and modeling approaches, the study identified the optimal input variable combination and the most effective model for arsenic concentration inversion in the study area. This research provides valuable scientific evidence and technical support for the application of hyperspectral remote sensing technology in monitoring soil heavy metal contamination.

#### 3.4.1. Modeling of Original Spectral Data

**Figure 4:**
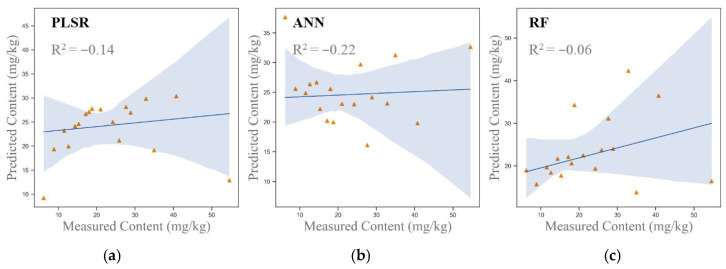
Scatter plots of original spectral data using three models.

The original spectral data were used for hyperspectral inversion modeling of arsenic concentrations in the study area, employing both linear models (PLSR) and nonlinear models (ANN and RF). The scatter plots of the inversion results are presented in Figure 4. The results revealed that the inversion accuracies of the PLSR, ANN, and RF models were −0.14, −0.22, and −0.06, respectively.

All three models demonstrated negative accuracy values, indicating significant overfitting. It may be caused by the small number of training samples. This outcome suggests that using only the original spectral data is insufficient for accurately predicting soil arsenic concentrations. The underlying issues may include noise, redundant information, or unresolved nonlinear relationships within the spectral data. To address these limitations, further optimization of the input variables or the inclusion of additional auxiliary data is required to improve the models’ generalization capabilities and prediction accuracy.

#### 3.4.2. Modeling of Original Spectral Data Combined with Arsenic-Correlated Soil Components

**Figure 5:**
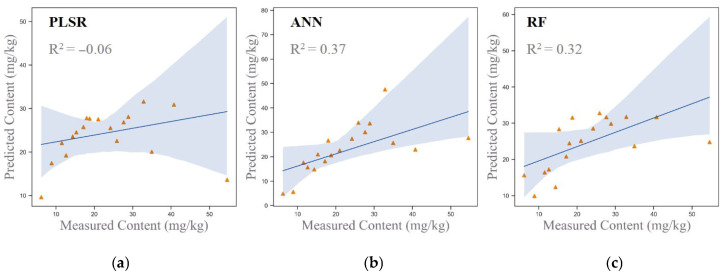
Scatter plots of original spectral data combined with arsenic-correlated soil components using three models.

To address the overfitting issue observed in the inversion of soil arsenic concentrations using only the original spectral data, this study integrated soil components significantly correlated with arsenic as additional input variables. These variables were combined with the original spectral data to enhance inversion accuracy. Using the combined dataset, inversion modeling was conducted with three models: PLSR, ANN, and RF. The scatter plots of the inversion results are shown in Figure 5.

The results revealed that the inversion accuracies of the PLSR, ANN, and RF models were −0.06, 0.37, and 0.32, respectively. Compared to the results obtained using solely original spectral data, the ANN and RF models exhibited substantial improvements in inversion accuracy after incorporating arsenic-correlated soil components. This improvement suggests that the inclusion of soil components effectively enhances the models’ predictive capabilities. The additional environmental information provided by the soil components compensates for the limitations of spectral data alone in characterizing arsenic concentrations. Such findings demonstrate the effectiveness of a multi-source data modeling approach that integrates spectral data and soil components, which provides valuable technical support for improving the accuracy and reliability of hyperspectral inversion of arsenic concentrations.

#### 3.4.3. Modeling of the Principal Component by PCA

**Figure 6:**
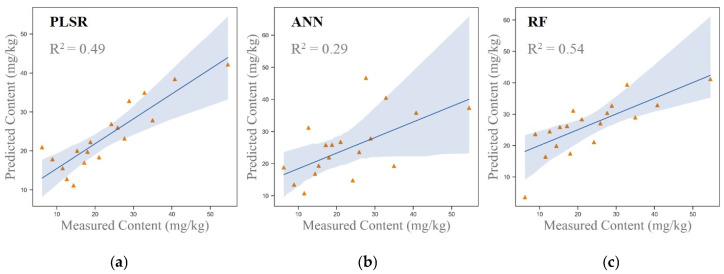
Scatter plots of principal components by PCA using three models.

One of the primary reasons for the poor performance of original spectral data in inverting heavy metal concentrations is the presence of severe collinearity issues and an excessive number of independent variables, a challenge extensively validated in prior research. To address this, the study applied dimensionality reduction to the original spectral data, leveraging PCA to mitigate collinearity and remove redundant information. The dimensionality-reduced spectral data were then used as input variables for the models. Inversion modeling was performed using three models: PLSR, ANN, and RF, with the results presented in Figure 6.

Figure 6 shows that the inversion accuracies of the PLSR, ANN, and RF models were 0.49, 0.29, and 0.54, respectively, with the RF model achieving the highest accuracy. The overall performance ranking was RF > PLSR > ANN. These findings highlight the significant improvement in model performance after PCA-based dimensionality reduction. The reduction in data dimensionality and the elimination of noise and redundant information enhanced the models’ generalization capabilities and prediction accuracy. This demonstrates the effectiveness of PCA in improving the reliability and robustness of inversion modeling for soil heavy metal concentration estimation.

#### 3.4.4. Hybrid Modeling of Principal Components and Arsenic-Correlated Soil Components

**Figure 7:**
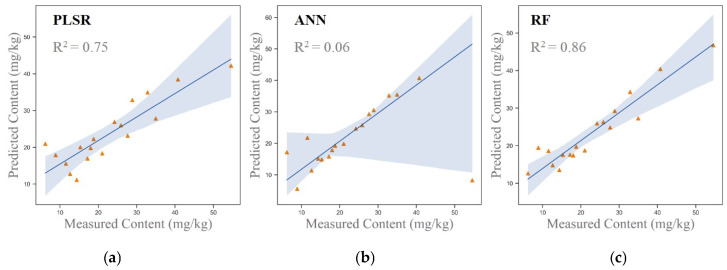
Scatter plots of PCs combined with arsenic-correlated soil components using three models.

By integrating soil components significantly correlated with arsenic and applying dimensionality reduction to spectral data, both of which have been shown to enhance model inversion performance, this study developed a new set of hybrid input variables. This approach reduces the dimensionality of the spectral data while retaining its essential spectral features and incorporates the additional environmental information provided by soil components. Together, these inputs were used to jointly invert soil arsenic concentrations. The scatter plots of the inversion results are shown in Figure 7.

The results in Figure 7 indicate that the hybrid input variables significantly improved inversion accuracy. The PLSR and RF models achieved accuracies of 0.75 and 0.86, respectively, successfully inverting arsenic concentrations in the study area. However, the ANN model performed poorly, with an inversion accuracy of only 0.06. This underperformance is likely due to the ANN model’s high sensitivity to outliers and errors in individual samples during the inversion process. These findings underscore the effectiveness of the multi-source data modeling approach, which combines spectral dimensionality reduction with soil components. This strategy markedly enhances the accuracy and reliability of arsenic concentration inversion, offering a robust framework for the application of hyperspectral remote sensing in soil heavy metal pollution monitoring.

## 4. Discussion

The arsenic (As) content in soil can be detected via hyperspectral data and is significantly influenced by various soil components. Specifically, under reducing conditions, sulfides react with arsenic to form insoluble arsenic sulfides (e.g., As_2_S_3_), which effectively immobilize arsenic. Iron oxides such as goethite and hematite, through adsorption or coprecipitation, also fix arsenic, leading to a significant correlation between their content and arsenic levels. Additionally, arsenic often coexists with heavy metals like cadmium (Cd) and chromium (Cr) in mineral deposits, with their concentrations showing positive correlations due to co-enrichment during mineralization. These soil components thus serve as indirect indicators of arsenic variability, acting as effective auxiliary variables in hyperspectral inversion of soil arsenic contamination and playing a crucial role in enhancing inversion accuracy [75].

In previous studies on the hyperspectral inversion of arsenic elements (Table 5), the focus has mostly been on the processing and analysis of hyperspectral data itself to establish the relationship between arsenic content and spectral characteristics. For instance, some studies [12,18] have improved the correlation between spectra and arsenic content through preprocessing of hyperspectral data, such as first-order differentiation and multiplicative scatter correction, before developing inversion models. The distinctive feature of this study lies in the integration of soil component information into the input variables for inversion. Certain soil components, such as iron oxides, have absorption characteristic bands that may correlate with arsenic content. By incorporating such soil component information into the input variables, this study effectively compensates for the singularity of spectral information, providing the inversion model with more data relevant to the target variable (arsenic content). This approach is expected to enhance the accuracy and reliability of the inversion. Notably, this mechanism has rarely been considered in previous hyperspectral inversion studies of arsenic elements, making it an innovative research approach.

PCA, whose effectiveness in spectral dimensionality reduction has been fully validated in previous studies [18,20], was applied to the original spectral data in this research. Seven principal components with eigenvalues > 1.0 were selected, accounting for a cumulative contribution rate of 99.9% (Table 4). These components retained key spectral information while significantly reducing data dimensionality, effectively resolving redundancy and severe collinearity inherent in spectral data. Additionally, soil components strongly correlated with arsenic (Cd, Cr, Cu, Ni, Pb, Zn, S, and T-Fe_2_O_3_) were incorporated as auxiliary input variables. This multi-source data fusion approach, integrating the merits of spectral dimensionality reduction and environmental information from soil components, notably enhanced the model’s performance in arsenic concentration estimation. These findings offer robust technical support for promoting the application of hyperspectral remote sensing in soil heavy metal contamination monitoring.

To evaluate the estimation performance of different input variable combinations across three models (PLSR, ANN, and RF), this study designed four input variable combinations: Combination 1 (original spectral data), Combination 2 (original spectral data combined with soil components significantly correlated with As), Combination 3 (principal components derived from dimensionality reduction), and Combination 4 (principal components combined with soil components significantly correlated with As). The results, as shown in Figure 6, indicate that the PLSR and RF models achieved their best performance with Combination 4, obtaining inversion accuracies of 0.75 and 0.86, respectively, confirming that the multi-source data fusion approach combining dimensionality-reduced spectra and soil components significantly enhanced model performance. In contrast, the ANN model exhibited poor performance across all combinations, with the highest inversion accuracy of only 0.32. This underperformance is likely due to the sensitivity of the ANN model to small sample sizes and its challenges in handling nonlinear relationships effectively. Overall, the RF model demonstrated superior performance in the context of multi-source data fusion, making it a reliable choice for hyperspectral inversion of arsenic concentrations.

The comprehensive comparison (Table 6) highlights that multi-source data fusion effectively addresses the issues of collinearity and redundancy in spectral data, leading to significant improvements in inversion accuracy. The outstanding performance of the PLSR and RF models with Combination 4 underscores the scientific value of integrating dimensionality-reduced spectral data with soil components. However, the limitations observed in the ANN model suggest the need for optimization of its network structure or the incorporation of regularization techniques to improve its performance.

In conclusion, this study confirms that integrating multi-source data related to soil arsenic can enhance the accuracy of arsenic content prediction. It not only provides a solid scientific basis and technical support for the application of hyperspectral remote sensing technology in soil heavy metal contamination monitoring, but also offers valuable insights into the effectiveness of multi-source data fusion and the significance of selecting appropriate inversion models. Meanwhile, several geographic parameters, such as distance to cities, slope gradient, distance to roads, and distance from pollution sources, may affect the spatial distribution of heavy metals [79]. Therefore, it is recommended that further research be conducted on the integration of hyperspectral technology with geostatistical methods based on geographic information systems (GIS), so as to further improve the prediction accuracy of arsenic content in agricultural soils [80]. Furthermore, the application of remote sensing data from different platforms, such as airborne and spaceborne hyperspectral data, represents a potential future direction for estimating soil heavy metals.

## 5. Conclusions

This study enhanced the accuracy and reliability of soil arsenic concentration inversion using hyperspectral remote sensing through the integration of multi-source data fusion, model performance optimization, and dimensionality reduction techniques. The exceptional performance of the RF model in multi-source data fusion and the effectiveness of PCA for dimensionality reduction offer valuable theoretical and methodological support for advancing hyperspectral remote sensing applications in soil heavy metal pollution monitoring. The key conclusions drawn from this study are as follows:

**(1) Significant Improvement in Inversion Accuracy through Multi-Source Data Fusion**

Combining dimensionality-reduced spectral data with these soil components (Combination 4) yielded the best results in both the PLSR and RF models, with inversion accuracies of 0.75 and 0.86, respectively. This highlights the scientific importance and practical potential of multi-source data fusion methods in enhancing inversion accuracy.

**(2) Optimal Performance of the RF Model**

The RF model consistently achieved higher inversion accuracy across all input variable combinations, particularly when multi-source data fusion was applied. This establishes the RF model as highly suitable for hyperspectral inversion of soil arsenic concentrations, offering robust and reliable performance.

**(3) Effectiveness of Spectral Dimensionality Reduction**

Principal Component Analysis (PCA) effectively addressed the challenges of redundancy in hyperspectral data. By retaining key spectral information while reducing dimensionality, PCA significantly enhanced model performance, providing essential technical support for processing hyperspectral data in arsenic concentration inversion tasks.

The results underscore the value of integrating advanced data processing techniques, such as multi-source data fusion and PCA, with robust modeling approaches like RF, to advance hyperspectral remote sensing technology for monitoring and assessing soil heavy metal contamination.

## Figures and Tables

**Table 1:** The analytical methods of soil elemental composition.

Elements	Analytical Methods
As	Determination of arsenic, antimony, and bismuth by hydride atomic fluorescence spectrometry
Cd	Determination of 32 trace elements by plasma mass spectrometry
Cr	Determination of 34 primary, secondary, and trace elements by X-ray fluorescence spectrometry
Cu	Determination of 32 trace elements by plasma mass spectrometry
Hg	Determination of mercury by cold vapor atomic fluorescence spectrometry
Ni	Determination of 32 trace elements by plasma mass spectrometry
P	Determination of 34 primary, secondary, and trace elements by X-ray fluorescence spectrometry
Pb	Determination of 32 trace elements by plasma mass spectrometry
SiO_2_	Determination of 34 primary, secondary, and trace elements by X-ray fluorescence spectrometry
Al_2_O_3_	Determination of 34 primary, secondary, and trace elements by X-ray fluorescence spectrometry
T-Fe_2_O_3_	Determination of 34 primary, secondary, and trace elements by X-ray fluorescence spectrometry
MgO	Determination of 22 elements by plasma optical emission spectrometry
CaO	Determination of 34 primary, secondary, and trace elements by X-ray fluorescence spectrometry
Na_2_O	Determination of 22 elements by plasma optical emission spectrometry
K_2_O	Determination of 34 primary, secondary, and trace elements by X-ray fluorescence spectrometry
pH	Determination of pH value of forest soil
SOM	Determination of total carbon and organic carbon by high frequency combustion-infrared carbon sulfur meter

**Table 2:** Statistical information for soil arsenic concentration (mg·kg^−1^) in the study area.

Arsenic Contamination (mg·kg^−1^)	Number	Mean	Max	Min	SD	CV
Calibration set	38	23.05	57.08	2.34	13.16	1.05
Validation set	18	22.95	54.57	6.14	12.26	0.53
Whole dataset	56	23.01	57.08	2.34	12.77	0.55

**Table 3:** Statistical results of soil properties (mg·kg^−1^) in the study area.

Soil Properties	Mean	Max	Min	SD	CV
Cd	0.64	2.11	0.04	0.40	0.62
Cr	65.24	116.91	10.55	25.19	0.39
Cu	89.04	320.86	21.68	64.48	0.72
Hg	0.11	0.41	0.02	0.07	0.63
Ni	24.77	47.98	5.70	10.46	0.42
Pb	65.28	592.44	17.30	75.66	1.16
Zn	135.47	401.09	47.43	65.64	0.48
P	850.30	3339.70	179.60	495.96	0.58
S	261.79	549.42	54.94	114.24	0.44
SiO_2_	65.63	76.62	7.45	9.83	0.15
Al_2_O_3_	14.39	24.48	1.88	3.28	0.23
T-Fe_2_O_3_	5.66	8.16	1.20	1.27	0.22
MgO	0.75	2.46	0.29	0.33	0.45
CaO	2.00	45.00	0.07	6.12	3.06
Na_2_O	0.50	2.28	0.05	0.44	0.89
K_2_O	1.88	2.78	0.31	0.41	0.22
SOM	38.66	75.97	6.66	18.57	0.48
pH	6.05	8.09	3.87	1.23	0.20

**Table 4:** Results of principal component dimensionality reduction analysis.

Components	Eigenvalue	Variance (%)	Cumulative Contribution Rate (%)
1	1847.74	92.34	92.34
2	77.84	3.89	96.23
3	46.67	2.33	98.56
4	16.54	0.83	99.39
5	7.48	0.37	99.76
6	1.52	0.08	99.84
7	1.11	0.06	99.90

**Table 5:** Prediction of soil arsenic contents using the reflectance spectroscopy of soils.

Sampling Site	Content Range (mg/kg)	Model	R^2^	Number of Samples	Authors
Agricultural regions	1.91–21.90	GA-PLSR	0.56–0.64	96	[76]
Agricultural area at mine	19.33–403.77	PLSR	0.58	33	[77]
Agricultural area at the Changjiang River Delta	6.13–13.30	PLSR	0.72	61	[78]
Agricultural area	10.25–133.36	GA-PLSR	0.42	94	[75]
Agricultural regions	2.34–57.08	RF	0.86	56	This study

**Table 6:** Compared the inversion performance of different input variables in the three models.

Input Variable	Model	R^2^	RMSE	RPD
Combination 1	PLSR	−0.14	12.74	0.96
	ANN	−0.22	13.14	0.93
	RF	−0.06	12.30	1.00
Combination 2	PLSR	−0.06	12.29	1.00
	ANN	0.37	9.49	1.29
	RF	0.32	9.80	1.25
Combination 3	PLSR	0.49	8.52	1.44
	ANN	0.29	10.03	1.22
	RF	0.54	8.11	1.51
Combination 4	PLSR	0.75	5.91	2.07
	ANN	0.06	11.55	1.06
	RF	0.86	4.45	2.75

## Data Availability

The author’s institution has strict requirements for data. If data is needed, please contact the author and provide it for use only after obtaining permission from the institution.
